# “Juggling” Behavior in Wild Hainan Gibbons, a New Finding in Nonhuman Primates

**DOI:** 10.1038/srep23566

**Published:** 2016-03-31

**Authors:** Huaiqing Deng, Jiang Zhou

**Affiliations:** 1 College of Life Sciences, Guizhou Normal University, Guiyang, Guizhou, 550001, China

## Abstract

Many species of primates use tools and manipulate objects. Environmental objects, such as sticks and branches, are used in locomotion, display, conflict, nesting, and foraging. This study presents observations regarding endangered male Hainan gibbons (*Nomascus hainanus*) selecting sticks and then throwing and catching them repeatedly. This act of Hainan gibbons was termed as “juggling” behavior. This study is the first record of branch use of this kind in free-living gibbons. While it is impossible to experiment on this only remaining population of Hainan gibbons, the deliberate acquisition and then throwing and catching of a stick raises myriad questions regarding their function. The study determined that the juggling behavior of Hainan gibbons, in the process of their brachiation, helps them accurately judge the distance and support strength of an object. It was also found that an adult individual’s proficiency in juggling behavior was much higher than that of a youth. Of all gibbon species, the juggling behavior of Hainan gibbon has a high degree of behavior refinement. Gibbons have the longest forearm than any other primates, which helps them in such performances—a unique mechanism that allows them to perform such unique activities, including juggling.

Object manipulation and tool use are common in wild primates^1^. Many species use branches and sticks to obtain resources^2^, facilitate communication, assist displays^3^, defend territories, attack intruders and predators, and aid movement on the ground^2^, in trees, and across water^4^. Gibbons are small arboreal apes that inhabit the rainforests of eastern and southeastern Asia^5^. Brachiation is the dominating locomotor behavior of wild gibbons^6^,^7^. It enables them to move quickly and efficiently through the forest canopy^6^.

The most comprehensive assessments of behavior, tool use, and culture are the products of a long-term study and observation^2^,^3^,^8^. In this study, the last remaining population of Hainan gibbons (*Nomascus hainanus*) was observed for over 10 years, and the number and diversity of documented behaviors continued to expand. Hainan gibbon is one of the most endangered primates and are restricted to a 12-km^2^ reserve in the tropical mountains of central Hainan^9^. Researchers of this study have been monitoring Hainan gibbons since 2000. There were 3 groups of Hainan gibbons and 6 solitary individuals in the reserve; a total of 26 individuals in 2013^10^. During the study period, one special behavior of the gibbons was noted in 2007–2010, which was never before observed in nonhuman animals in the wild. This behavior is reported in the present study and was termed as “juggling” because of its apparent deliberate and clearly defined nature.

## Results

“Juggling” is a form of stick throwing and catching behavior in wild Hainan gibbons. This study presents the observations of only one group of gibbons comprising an adult male (BM1), two adult females (BF1, BF2), a subadult male (BM4), and two juvenile males (BM5 and BM6). Juggling for five times continuously was observed only in the males of this group.

Observation 1: On December 3, 2007, at 7:25 AM, the adult male BM1, while hanging, deliberately broke off a 50- to 60-cm stick (diameter 3 cm) and threw it once above his head. The branch descended and hit his shoulder and then fell to the forest floor. BM1 was in the immediate presence of BF1, BF2, BM4, and BM5, who appeared to be observing him.

Observation 2: During routine observations on October 3, 2008, the juggling behavior of BM1 was again observed. From 8:50–8:53 AM, BM1 foraged on fruits from an arbor tree (*Microcos chungii*). He then ascended to the canopy (average height 18 m) and engaged in a short bout of self-grooming. After 1 minute, while hanging from his right hand, he used his left hand to deliberately break off a stick 40- to 50-cm long (diameter 3–4 cm), and threw it 1–1.5 m above his head. He then caught the falling branch with the same hand and repeated this action 10 times before carrying the stick in his mouth to another location (Movie S1). BF1, BF2, BM4, and BM5 were sitting 10- to 12-m away and appeared to be observing BM1. No vocalizations were heard.

Observation 3: On July 30, 2008, at 10:25 AM, the juvenile male BM5sitting on a tree threw a 10-cm piece of bark acquired from the tree (*Endospermum chinense*) into the air and caught it. He repeated this action three times (Movie S2). No group members appeared to be observing BM5, although animals were 5–6 m away.

Observation 4: On September 2, 2010, the subadult male BM4 sitting on a tree broke off a 15- to 18-cm stick and threw it in the air and caught it, repeating the action three times.

Observation 5: On September 6, 2010, the juvenile male BM6 sitting on a big branch acquired a 12- to 15-cm piece of bark from a tree and threw it into the air and caught it, repeating the action two times.

## Discussion

The definition of tool use proposed by Beck^11^ has dominated behavioral research for 30 years. Central to Beck’s classification was the use of an unattached object to alter the state or condition of another object, or an organism, or the tool user itself, when the individual holds the tool immediately prior to use. More recently, St Armant and Horton^12^ extended Beck’s ideas to include the use of tools to make a mechanical dynamic connection between the user and an object, and to affect the transfer of information between the user and the environment or other animals. Juggling involves detaching an object (stick), which could be regarded as tool use under both the aforementioned and other definitions^1^. However, inherent in both definitions is a reference to the goal or intention of the tool user. The small number of records of the juggling behavior in Hainan gibbons limited the certainty with which to address their motivation, but it was clear that the behavior was deliberate and repeatable and that the stick was obtained from the environment to perform a specific and deliberate function. For example, the stick was deliberately snapped off and used in juggling, and, further, after one bout of juggling, a male was observed to carry the stick away in his mouth, perhaps for later use as other primates are known to do with some tools^13^.

Sticks and branches are used as tools in locomotion, display, and feeding by wild chimpanzees (*Pan troglodytes*)^2^, orangutans (*Pongo pygmaeus abelii*)^3^, and gorillas (*Gorilla gorilla*)^4^, and dropped and thrown by many primates^1^. However, acquiring a stick, or any object, and then throwing it with the intention of catching it remains unreported, and is perhaps the first example of tool use of this kind in a nonhuman animal in the wild. There is a single report of two male gibbons engaging in spontaneous throwing and catching of a colored ball and food items at a research facility in Japan^14^. These individuals have also been observed to throw food and catch it in the mouth. Given the captive environments in which these two gibbons live and the mostly man-made objects they throw, caution is needed while comparing the behavior of these animals with those observed in wild animals in this study.

In the present study, observations of this behavior lack experimental validation but are nevertheless important for guiding future research and hypotheses. Gibbons are well known for advanced hand–eye coordination during locomotion, and it is possible that within this advanced cognitive and mechanistic context, juggling has evolved. It is also possible that this skill has been transferred from a mechanistic-locomotion context to a sociocultural context, and may qualify under the definitions of a unique cultural variant of play or display behavior. Since juggling appears to be unconnected to feeding or locomotion, it may function as a male display. Juggling was observed only in males, and group members appeared to observe juggling when done by the dominant male BM1 and not by the juveniles BM5 and BM6. Given the degree of coordination necessary to juggle a stick, this behavior may function as a form of male display and communicate information to others. Juggling was deliberate and performed with intent. The sticks were detached from the trees, a behavior in itself unusual in the gibbon species. The stick was then thrown into the air and close to the individual so that it can be caught and thrown again. Both these steps indicate that the gibbon intended to use the stick in a specific manner that included repeated throwing and catching. This raises the notion that the individuals have a sense of what juggling looks like and how it is performed; otherwise, the stick would just fall to the ground and form part of the background. Therefore, it seems that juggling requires advanced cognitive skills and an idea of what juggling is before it can be performed precisely.

Playing behavior is very important in developing and improving various functional skills in an individual as can be seen from earlier observations, such as identifying an object, analyzing its use, experimenting with it, studying it more closely, controlling it by throwing and catching again, which in turn help them adapt to the natural environment. The playing behavior in mammalians is imperative for the development of social behavior. More intelligent and sociable species play more elaborately. And social play helps improve the physical quality to strengthen sports skills and improve survival skills^15^,^16^. The importance of learning and playing for animals using tools is unassailable. Schiller found that if individuals are forbidden to use sticks to play, their ability to use tools to solve problems will be significantly reduced^17^.

**Figure 1:**
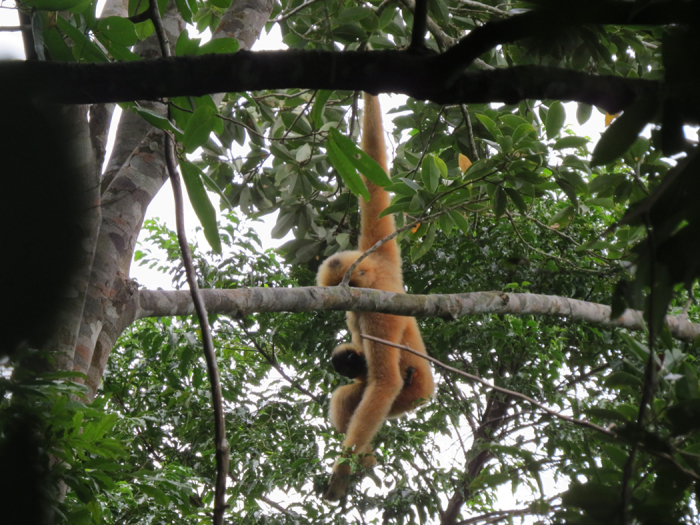
Brachiation of an adult Female Hainan Gibbon with her infant (study animals, photo provide by Huaiqing Deng).

Rapid brachiation is inherently more dangerous than other locomotor modes, such as climbing. Brachiation requires rapid and dexterous maneuvering, puts increased strain on supports, and is associated with an increased risk of failure, either by the animal or by the support^18^,^19^. Support size significantly affected the type of locomotor behavior employed by gibbons; branches were most frequently (62.4%) used in *N. nasutus*^20^. The juggling behavior plays an important role in judging which branches have the ability to support the fast movement of Hainan gibbons. In the present study, it was found that an adult individual’s proficiency in juggling behavior was much higher than that of a youth. The juggling behavior of Hainan gibbons, in the process of its brachiation (Fig. 1), helps them to accurately judge the distance and the support strength of an object, and has a very important biological significance. This playing behavior of Hainan gibbons, throwing an object up and catching, and then seizing practice, helps them to use tools existing in the environment, such as branches, to assist them in moving quickly and efficiently through the forest canopy. The study agrees that the more intelligent the species, the better the play. The juggling behavior of Hainan gibbon has a high degree of behavior refinement. These unique characteristics of brachiation and acrobatic movement of the creatures may be due to the functional morphology and specific adaptation of arm structure in gibbons. Gibbons have the longest forearm than any other primates, which plays an important role in such performance—a unique mechanism that allows them to perform alternative unique activities, including juggling.

Anecdotal observations of novel behaviors in wild animals can challenge existing concepts of tool use, generate testable hypotheses, and guide future research. For example, could juggling be a form of play, display, or communication? Further observations of this endangered species are required. The researchers of this study will continue their monitoring of this species. Unfortunately, the opportunity to study species of interest such as Hainan gibbons is jeopardizing because of continued threats to the survival of their very small population.

## Methods

In this study, two remaining family groups of this species were being tracked since 2002. These two groups were semi-habituated to the presence of the researchers of this study. So it was often possible to conduct observations directly from the forest floor. It was possible to identify individuals according to their differences in size and pelage color. The animals were located with their morning calls (in which all group members except the juveniles participated) from three listening posts staffed from 5:30 AM. The researchers then moved out toward the animal group and commenced observations prior to sunrise and terminated at approximately 4:30 PM. During observations, the vertical (14–28 m, varies with canopy height) and horizontal (usually 0 m) distances of the researchers from the animals, the height of the animals in the canopy, and the relative distance between the group members were recorded using binoculars or video cameras. Singing, grooming, feeding, traveling, playing, foraging, and encounters between the two family groups were also recorded using focal animal and all occurrence sampling. The time and duration of each behavior, the identity of the focal animal, and whom the behavior was directed were noted. The location of the animals and all group members during each behavior was also recorded. Digital video cameras (Canon i850) were often used, along with a digital camera (Sony DSC-HX100). GPS records and data on precipitation, temperature, relative humidity, and the time of sunrise and sunset were also noted. The animals were photographed frequently and extended recordings using a digital video camera were often made.

The methods were carried out in accordance with the Bawangling National Nature Reserve Administration approved guidelines. All experimental protocols were approved by Bawangling National Nature Reserve Administration. No human participants, specimens, vertebrate animals, embryos, or tissues were used in this study.

## Additional Information

**How to cite this article**: Deng, H. and Zhou, J. "Juggling" Behavior in Wild Hainan Gibbons, a New Finding in Nonhuman Primates. *Sci. Rep.* **6**, 23566; doi: 10.1038/srep23566 (2016).

## Supplementary Material

Supplementary Information

Supplementary Movie S1

Supplementary Movie S2
