# Supplementary material for: “Juggling” Behavior in Wild Hainan Gibbons, a New Finding in Nonhuman Primates
Source: Sci Rep. 2016 Mar 31;6:23566. doi: 10.1038/srep23566 (PMC4814837; doi:10.1038/srep23566)
Supplement: Supplementary Information [file srep23566-s1.pdf]

‘Juggling’ Behaviour in Wild Hainan Gibbons, a New Finding in Nonhuman Primates

Huaiqing Deng & Jiang Zhou*

Movie S1. This video shows a male Hainan gibbon (BM1) engaged in juggling behaviour. Filming was conducted from ground level; the male is approximately 16 m high. The first part of the movie shows the male breaking off and then juggling a stick for ten times, later the camera pans to show two of the individuals leaving after the juggling (MPG format, 6.99MB, 42 s).

Movie S2. This video shows a juvenile male Hainan gibbon (BM5) engaged in juggling behaviour. Filming was conducted from ground level. Bark is thrown three times (MPG format, 1.83 MB, 11 s).
